# A Case of Quadruple Birth

**Published:** 1854-11

**Authors:** S. Kennerly

**Affiliations:** Augusta county, Virginia


*A Case of Quadruple Birth.* By S. Kennerly, M. D., Augusta county, Virginia.—On the night of the 5th of August, I was called to see Mrs. W., supposed to be in labor. On my arrival I was informed by my friend, Dr. A. Waddell, who had also been called in, that he had just delivered the head of a child, which had been expelled feet foremost, the head being retained. He was told that it had been in that position for two hours. Upon examination, we found that there was a second child inclosed in its membranes; and as there had been no recurrence of pain since the partial expulsion of the first child, and the mouth of the womb being entirely relaxed, we concluded that the safest plan would be to administer ergot, and complete the delivery. We accordingly gave her ʒi of liquor ergotina, prepared by Purcell, Ladd & Co., which quickened and increased her pain; and in three quarters of an hour, the second child was expelled, followed by a subsidence of pain. On examination, we found a third child presenting, breech foremost, with no membranes around it. After waiting half an hour and no pain recurring, we brought down the feet, and repeated the liquor ergotina in the dose of ʒss, and in half an hour, the third child was expelled. On examination, we found a fourth child presenting, head foremost, which was expelled in about twenty minutes, inclosed in its proper membranes. There were two distinct placentas, and they soon followed the birth of the fourth child. One was very large, and divided into three distinct lobes, each lobe having its respective chord attached to its centre. The smaller placenta was a little larger than either lobe of the larger placenta. The children (three boys and a girl) were all born alive, but neither lived over fifteen minutes. I suppose they were not viable, for they were evidently born too soon, though they were perfect in every respect, so far as I could judge. The finger and toe nails were perfect. They weighed nearly 8 pounds next morning, and measured 16 inches in length. Mrs. W. did not expect to be confined until the latter part of October next, and thinks she quickened about the middle of June. So the children must have been under seven months. She did not suffer as much as in either of two former labors of single births, and is doing remarkably well. What seems a little singular is, that there should have been a total suspension of uterine contractions for two hours after expulsion of the first child, even while its head remained in the vulva, and the second child protruding from the womb into the vagina. I think that the second and third children were inclosed in the same membranes, though their cords were each attached to its respective lobe of the larger placenta. There was but little hemorrhage, and the womb contracted well after the expulsion of the placentas.—*Stethoscope.*

